# Body Mass Changes Across a Variety of Running Race Distances in the Tropics

**DOI:** 10.1186/s40798-016-0050-3

**Published:** 2016-07-19

**Authors:** Desmond Wei Tan, Si Hui Yap, Mingchang Wang, Priscilla Weiping Fan, Ya Shi Teo, Priathashini Krishnasamy, Lingaraj Krishna, Tamara Hew-Butler, Jason Kai Wei Lee

**Affiliations:** 1 Yong Loo Lin School of Medicine, National University of Singapore, Singapore, Singapore; 2 National University Hospital Sports Centre, National University Health System, Singapore, Singapore; 3 Defence Medical & Environmental Research Institute, DSO National Laboratories, Singapore, Singapore; 4 Department of Orthopaedics, Tan Tock Seng Hospital, Singapore, Singapore; 5 School of Health Sciences, Oakland University, Rochester, MI USA; 6 Lee Kong Chian School of Medicine, Nanyang Technological University, Singapore, Singapore

## Abstract

**Background:**

Current literature evaluating body mass (BM) changes across a variety of running race distances is limited. The primary objective of this study was to profile the range of BM changes across race distances. The secondary objective was to evaluate the prevalence of exercise-associated hyponatremia (EAH) in runners admitted to the on-site medical tent following participation of race events of different distances.

**Methods:**

A total of 1934 runners across seven footrace categories (10-, 21-, 25-, 42-, 50-, 84-, and 100-km) were included in the study. One thousand eight hundred eighty-seven runners had their BM measured before and after each race. Blood sodium concentrations were measured from the remaining 47 symptomatic runners admitted to the on-site medical tents and did not complete the race.

**Results:**

In terms of hydration status, 106 (6 %) were overhydrated, 1377 (73 %) were euhydrated, and 404 (21 %) were dehydrated. All race distances exhibited similar percentage of overhydrated runners (5 % in 10 km, 3 % in 21 km, 5 % in 25 km, 6 % in 42 km, 8 % in 50 km, 7 % in 84 km, and 6 % in 100 km). Forty-seven runners were admitted to the medical tents. Eight (17 %) were diagnosed with EAH (4 from 42 km, 2 from 84 km, 2 from 100 km), 38 (81 %) were normonatremic, and 1 (2 %) was hypernatremic. The % ΔBM across all races ranged from −8.0 to 4.1 % with a greater decrement noted in the 42-, 50-, 84-, and 100-km categories.

**Conclusions:**

Approximately 3–8 % runners had increased post-race BM, suggesting overhydration regardless of race distance. Symptomatic EAH was seen at race distances at or above 42 km, where BM changes demonstrated the widest range of values.

## Key Points

In contrast to common beliefs, amongst those admitted to the on-site medical tents in tropical races, there seemed to be more cases of overhydration than dehydration.

Relevant authorities and stakeholders should relook at available evidence and develop a more rational and pragmatic fluid replacement strategy, aimed at optimizing, rather than maximizing fluid intake.

## Background

Changes in body mass are often used as a surrogate measure of estimating sweat water losses during exercise, serving as an individualized fluid replacement guide during exercise in a variety of environmental conditions [1]. However, it has been previously shown that male and female runners underestimate sweat losses by roughly 50 % after a 60-min run in the heat [2]. Such underestimations of fluid replacement needs have sparked recommendations suggesting that athletes drink above the dictates of thirst, which has resulted in an overestimation of fluid requirements, weight gain, and fluid overload hyponatremia [3]. Exercise-associated hyponatremia (EAH) typically occurs during or up to 24 h after prolonged physical activity and is defined by a serum or plasma sodium concentration below 135 mmol/L [3]. The principal mechanism of EAH has been postulated to be excessive fluid consumption (i.e., overhydration) beyond the capacity for renal excretion due to the failure of suppression of antidiuretic hormone (ADH), leading to resultant body mass (BM) gain and eventual hyponatremia [3–5]. However, not all changes in body mass directly represent total body water losses or gains. While insensible losses from sweating and respiration, as well as oxidation of fuel substrates such as glycogen lead to mass loss, oxidative breakdown produces metabolic water, increasing body water and causing changes in body mass as well [6, 7]. Up to 3 % of body mass may be lost without changes in total body water, effectively allowing the body to remain in a state of euhydration [6, 7]. Hydration states determined based on % ΔBM have thus been classified as “overhydration” > 0 %, “euhydration” −3 to 0 %, and “dehydration” < −3 % [8]. A negative linear relationship between serum sodium levels and changes in body mass has been established, substantiating the growing evidence that increasing body mass gain could potentially increase the risk of EAH [9]. In spite of this, a study by Hoffman et al. [10] revealed that of EAH with dehydration as measured by mass loss was more predominant than EAH with overhydration as measured by mass gain in 161-km ultramarathoners in Northern California. The difficulty in establishing a consistently predictable relationship between EAH and body mass changes is likely due to the interplay of multiple factors that influence both mass changes and the prevalence of EAH. Other risk factors reported to cause EAH include prolonged exercise exceeding 4 h, low pace racing, low body mass, female gender, and hot environments [3, 11].

The current literature for EAH in Asia is limited. Moreover, there is increasing evidence to suggest that EAH may even occur in much shorter distance events such as half-marathons and sprint races taking approximately 90 min [12, 13]. Dehydration may be falsely perceived to be of greater concern in comparison to overhydration during endurance events in the tropics, and the behavioral response may be to drink copiously. As such, our previous EAH study [14] reported the first cases of EAH in Asia with a prevalence of 38 % amongst symptomatic runners admitted to the medical tent. Significant increases in mean BM were noted in 7–8 % of the 417 runners after a 42- and 84-km ultramarathon in the night. Thus, in contrary to previously held beliefs, concerns over overhydration and EAH should be regarded as important as dehydration during organized sporting events in tropical climates, such as in Singapore.

The current study builds upon the previous work by Lee et al. [14] with the addition of (1) a larger sample size to increase statistical power; (2) inclusion of daytime running events which expose runners to higher environmental temperatures; and (3) inclusion of other race distances, such as the increasingly popular 10- and 21-km races, to more critically evaluate changes in BM and the incidence of EAH across a broader range of events held in Asia. The primary objective was to profile the runners’ hydration status via changes in BM from pre- to post-race. The secondary objective of this study was to evaluate the prevalence of EAH in athletes admitted to on-site medical tent following participation of a running event under our local tropical climatic conditions.

## Methods

### Study Population and Setting

In this National University of Singapore Institutional Review Board-approved study, we analyzed participants across three different race events: (1) Adidas Sundown Marathon (ASM) 2009—42- and 84-km categories; (2) The North Face (NF) 100 Race 2009—25-, 50-, and 100-km categories; and (3) Standard Chartered Marathon (SCM) 2009—10-, 21-, and 42-km categories. The estimated numbers of runners in each of these three races were 11,000, 1000, and 60,000, respectively. The ASM was a night event while the NF and SCM were daytime events. All races were conducted on a relatively flat ground course in the city area and seaside park with the exception of NF, which involved off-road trail running in a largely undulating terrain. For the NF, runners were told to bring their own race equipment (hydration pack, belt, or bottle). There were four official fluid stations, one at the starting/ending point and three along the race route and three additional unofficial water points which can be found along the race route. For the ASM and SCM, fluid stations were positioned at approximately 2–3 km intervals. Sports drinks (100Plus, Fraser and Neave Limited, Singapore; 248 kcal/L, carbohydrate 62 g/L, sodium 20.9 mmol/L, potassium 3.4 mmol/L) and water (Ice Mountain Mineral Water, Fraser and Neave Limited, Singapore) were provided at the stations. Climatic conditions (temperature, relative humidity, and wind speed) on the day of respective races were collected from the local weather station. 

### Outcome Measurements

Body mass measurement was offered by the medical race director at each of these races, and that these measurements were fully voluntary. Pre-race body mass were recorded within an hour before the commencement of the race while post-race body mass were recorded immediately after completion. These were obtained in the same racing attire within 10 m after crossing the finishing line to avoid fluid consumption before measurement. A digital platform balance (BBA211, Mettler Toledo, Germany or Seca, Hamburg, Germany) with an accuracy of 0.1 kg was used for all races. The percentage change in body mass (% ΔBM) for each runner was calculated using the following formula: [(post-race body mass − pre-race body mass)/pre-race body mass] × 100. Hydration states determined based on % ΔBM were thus classified as “overhydration” > 0 %, “euhydration” −3 to 0 %, and “dehydration” < −3 % [8, 10]. For symptomatic runners admitted to the on-site medical tent, intravenous cannulation was performed by physicians and 3 mL of blood was collected as part of the standard medical protocol. Symptomatic runners included runners who experienced nausea, vomiting, confusion, headache, seizures, or acute respiratory distress, which may be symptoms of EAH [8]. On-site analysis was immediately performed for sodium, potassium, chloride, blood urea nitrogen, glucose, hematocrit, and hemoglobin levels using a hand-held i-STAT blood analyzer (i-STAT 6+ cartridge, 06F05-01; i-STAT System, Abbott Point of Care, NJ). This was part of the medical standard operating procedure performed for symptomatic runners. The reliability of the hand-held i-STAT blood analyzer has been established by comparison with a standard laboratory electrolyte analyzer [15]. Plasma concentrations of sodium were used to classify results as “hypernatremia” > 145 mmol/L, “normonatremia” 135 to 145 mmol/L, and “hyponatremia” < 135 mmol/L [8]. Body mass measurements of symptomatic runners who presented to the medical tent were, unfortunately, not obtained due to significant clinical symptomatology that precluded ambulation to designated weighing stations.

### Statistical Analysis

Normality of data was assessed using the Shapiro Wilk test. A paired *t* test was used to compare pre-race and post-race body mass while an independent *t* test with Bonferonni correction was used to compare % ΔBM between different race distances. Statistical significance was set a priori at *p* < 0.05. A Pearson’s product moment correlation coefficient (*r*) was used to compare the relationship between finishing time and % ΔBM. The climatic data were logged and analyzed every minute. All data in this study are presented as mean (SD) unless otherwise stated. All statistical analyses were performed using Statistical Package for the Social Sciences (SPSS) Version 22.0 (SPSS Inc, Chicago, IL, USA).

## Results

### Study Population

Body mass measurements were collected from 1575 (81 %) men (M) and 359 (19 %) women (F) runners. The breakdowns for each race category are as follows: 191 (149 M, 42 F) in 10-km; 193 (147 M, 46 F) in 21-km; 591 (451 M, 140 F) in 25-km; 535 (467 M, 68 F) in 42-km; 281 (229 M, 52 F) in 50-km; 123 (114 M, 9 F) in 84-km; 20 (18 M, 2 F) in 100-km. Additional 47 runners were admitted to the on-site medical tents following the participation of race events.

### Climatic Conditions

The climatic conditions of the three respective race events were recorded and compared (Table 1). Parameters remained relatively constant and similar across all races.

**Table 1:** Climatic conditions of the profiled race events. Data are presented as mean with standard deviation shown in parentheses

Parameter	Adidas Sundown Marathon	Standard Chartered Marathon	North Face 100 Run
Dry bulb (°C)	29.0 (1.9)	28.6 (2.2)	30.1 (3.2)
Wet bulb (°C)	24.4 (1.5)	25.6 (1.3)	26.4 (1.3)
Globe (°C)	34.9 (8.2)	34.5 (7.5)	35.1 (8.3)
Relative humidity (%)	89 (7)	69 (19)	68 (23)
Wind speed (m/s)	0.3 (0.2)	0.7 (0.6)	0.5 (0.5)

### Body Mass Changes

Mean changes in body mass for 1887 runners who completed the race based on pre- and post-race values were reported for each category in Table 2. All categories reported significant decreases in mean body mass (*p* < 0.001). In comparison of % ΔBM across categories, the 42-, 50-, 84-, and 100-km races showed a significantly greater decrement (*p* < 0.05) in mean % ΔBM than the 10-, 21-, and 25-km races. A direct weak correlation was found between finishing time and % ΔBM for the 10- and 25-km races (Table 3). No correlation was observed for the other race categories. BM was not measured in the additional 47 symptomatic runners who were admitted to the on-site medical tents.

**Table 2:** Changes in pre- and post-race body mass in all races (*p* < 0.001). Data are presented as mean with standard deviation shown in parentheses

Category	Pre-race body mass (kg)	Post-race body mass (kg)	Change in body mass (kg)	Range of % ΔBM
10-km (*N* = 184, % = 9.8)	67.0 (11.7)	66.9 (11.6)	−0.8 (0.5)	−3.0–1.4
21-km (*N* = 181, % = 9.6)	66.6 (10.3)	66.0 (10.3)	−1.3 (0.8)	−5.6–1.6
25-km (*N* = 591, % = 31.3)	66.3 (11.9)	65.0 (11.0)	−1.3 (0.9)	−6.0–4.1
42-km (*N* = 522, % = 27.7)	66.4 (9.3)	65.3 (9.3)	−1.3 (1.0)	−7.8–2.7
50-km (*N* = 281, % = 14.9)	65.3 (9.7)	63.8 (9.5)	−1.6 (1.2)	−7.8–2.8
84-km (*N* = 110, % = 5.8)	64.3 (8.7)	62.9 (8.7)	−1.4 (1.1)	−8.0–1.4
100-km (*N* = 18, % = 1.0)	66.2 (9.1)	64.1 (8.9)	−2.1 (1.3)	−5.6–1.6

**Table 3:** Correlation between finishing time and % ΔBM (represented by *r* value). Finishing time in minutes depicted as mean with standard deviation shown in parentheses

	Category	Finishing time (min)	*r* value	*p* value
10-km	Total	73 (14)	0.227	<0.05
	Men	71 (13)	0.188	<0.05
	Women	81 (16)	0.339	<0.05
21-km	Total	149 (27)	−0.008	0.916
	Men	146 (26)	0.006	0.949
	Women	158 (25)	−0.129	0.397
25-km	Total	213 (32)	0.381	<0.001
	Men	213 (32)	0.376	<0.001
	Women	213 (32)	0.405	<0.001
42-km	Total	321 (64)	0.075	0.262
	Men	322 (65)	0.064	0.372
	Women	311 (52)	0.121	0.530
50-km	Total	456 (57)	0.047	0.430
	Men	457 (58)	0.087	0.188
	Women	451 (51)	−0.140	0.324
84-km	Total	721 (93)	−0.040	0.262
	Men	726 (94)	−0.092	0.273
	Women	668 (92)	0.062	0.350
100-km	Total	869 (114)	0.244	0.330
	Men	870 (84)	0.397	0.128
	Women	869 (119)	1.000	<0.001

There were only two women runners in the 100-km race

### Hydration Status

The % ΔBM across all races ranged from −8.0 to 4.1 %. In terms of hydration status, as defined by percent changes in body mass, 106 (6 %) were overhydrated (% ΔBM > 0), 1377 (73 %) were euhydrated (% ΔBM between −3 and 0), and 404 (21 %) were dehydrated (% ΔBM < −3). The breakdown of distribution of % ΔBM across the different races is shown in Table 4 and Fig. 1. Of note, the races exhibited similar percentages (3–8 %) of overhydrated runners regardless of distance covered. Sub-analysis by gender showed similar percentage of overhydration between the women runners (7 %; 24 out of 354) and their men counterparts (6 %; 91 out of 1533; *p* = 0.345).

**Table 4:** Distribution of changes in % ΔBM across races (% in parentheses)

Category	Number of runners	Dehydration % ΔBM < −3	Euhydration % ΔBM −3 to 0	Overhydration % ΔBM > 0
10-km	184	0 (0)	175 (95)	9 (5)
21-km	181	20 (11)	155 (86)	6 (3)
25-km	591	133 (23)	427 (72)	31 (5)
42-km	522	111 (21)	382 (73)	29 (6)
50-km	281	95 (34)	164 (58)	22 (8)
84-km	110	33 (30)	69 (63)	8 (7)
100-km	18	12 (67)	5 (28)	1 (5)

**Fig. 1:**
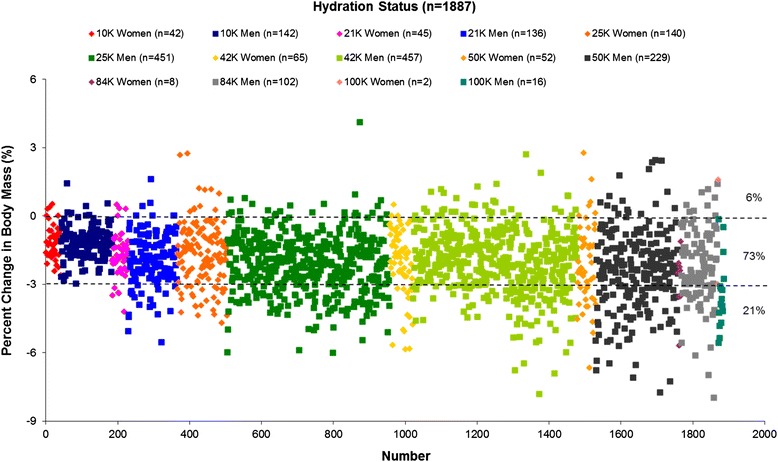
Distribution of changes in % ΔBM across races

### Exercise-Associated Hyponatremia

Forty-seven runners were admitted to the on-site medical tents for symptoms such as confusion, nausea, vomiting, bloating, and/or puffiness. Blood samples were collected and analyzed. Eight (17 %) were hyponatremic, 38 (81 %) were normonatremic, and 1 (2 %) was hypernatremic. The results of the blood analysis of the eight runners (7 M, 1 F) diagnosed with EAH are shown in Table 5. The incidence of EAH was found only in the 42-, 84-, and 100-km categories.

**Table 5:** Blood analysis of runners diagnosed with symptomatic EAH treated within the medical tent. Normal ranges of values and units shown in parentheses

Category	Gender	Na^+^ (135–145 mmol/L)	K^+^ (3.5–5.0 mmol/L)	Cl^−^ (96–106 mmol/L)	BUN (7–20 mg/dL)	Glu (4.0–11.0 mmol/L)	Hct (35–50 %)	Hb (12.0–17.5 g/dL)
42-km	Male	134	4.1	104	21	7.1	44	15.0
42-km	Male	134	6.1	104	16	5.1	58	19.7
42-km	Male	134	6.1	104	16	5.1	58	19.7
42-km	Female	132	3.6	98	14	6.1	42	14.3
84-km	Male	131	4.3	101	24	9.2	40	13.6
84-km	Male	117	5.6	90	20	6.5	45	15.3
100-km	Male	127	4.2	98	25	7.0	49	16.7
100-km	Male	127	4.5	93	39	5.5	44	15.0

## Discussion

The mean decrease in BM across all races in this study, with a greater magnitude change as the race length increases, is not unexpected. Mass loss from oxidation of metabolic substrates for energy production and fluid losses from sweat inevitably leads to a decrease in BM, and this loss is amplified by greater energy requirements from increasing race durations and distances [7]. However, sub-analysis in the breakdown of ranges of % ΔBM revealed a wide range of −8.0 to 4.1 %, and this is comparable with those previously reported in endurance running events [6, 8, 16]. The presence of a positive change in BM highlights runners who gained BM during the race. The concept of excessive fluid consumption leading to overhydration with resultant body mass gain has been well established [8]. In this study, 6 % (106/1887) of runners appeared to drink beyond fluid excretion rates with a slightly higher incidence of body mass gain noted in the longer distance categories (42-km and above). Excessive concern over dehydration and hyperthermia developing during exercise in tropical climates may have led to overzealous fluid consumption in our runners, similar to other ultraendurance races [5, 10, 17]. In addition, overdrinking behavior could have been reinforced by companies selling sports drinks [18, 19] despite the fact that all sports drinks are hypotonic with regard to sodium and potassium content [3].

However, even with excessive consumption of fluid, mass gain should not normally occur during exercise [7]. The excretory capacity of kidneys is between 750 and 1440 mL/h and the rate of sweating estimated to be 500 mL/h [20]. Thus, even in slow runners, fluids may be safely ingested in excess of 1.5 L/h without the development of water retention. The mechanism of mass gain during exercise has been attributed to the failure of antidiuretic hormone (ADH) suppression, which leads to a reduction in the excretion of free water by the kidneys [21]. There is increasing evidence suggesting several non-osmotic stimuli that can lead to a submaximal suppression of ADH during exercise, including intense exercise, nausea, and/or vomiting, plasma volume contraction, hypoglycemia, pain, and other yet-to-be determined factors [3, 22–24].

Relationships between race time and hydration levels have previously demonstrated a positive association between finishing time and % ΔBM (as a surrogate for hydration status) [25]. Our study weakly supported these findings. This is in contrast with the current literature which demonstrates that runners participating in longer distances (42-km and beyond) are at a greater risk of overhydration [8, 10, 14, 17, 16, 26–28]. This discrepancy may result from other variables not studied, such as the experience levels of the runners taking part in these shorter races. Slow pace running might have also contributed to lower sweat rate and longer duration for fluid consumption, increasing the risk of overhydration [3, 11, 24]. In our study, we also noted a comparatively lower proportion of runners who were dehydrated (loss of BM post-race) in the shorter race distances (0 % for 10-km and 11 % for 21-km). This contrasts to a higher incidence of dehydration in longer race distances that ranged from 21 to 67 %. It is postulated that increasing race distance and thus race duration contributes to a higher degree of dehydration, which could account for why the shorter distance runners did not face a significant issue of dehydration. Therefore, while post-race BM loss increases in longer race distances due to increased substrate oxidation, it is noteworthy that increased BM could instead occur due to reasons aforementioned. While the current study is unable to critically evaluate the relationship between race time and changes in body mass, it does establish the foundation for future biochemically based investigative work across a wider variety of running distances.

In this study, we reported eight cases of EAH diagnosed based on clinical symptoms and biochemical verification of EAH in runners who had presented to the medical tent. However, the actual incidence may be higher, as runners who had not presented to the medical tent would not have been captured in this group. While the prevalence of symptomatic EAH may appear low, it should be emphasized that EAH accounted for 17 % of all cases that presented to the medical tent. The majority of the current literature on EAH focused on longer distance races, such as ultra-marathons. Recent evidence, however, has documented the occurrence of symptomatic EAH in much shorter duration and distance events such as half-marathons and sprint races [12, 13]. As such, we augment the current literature by providing the largest study to date with the inclusion of a wider range of shorter race distances (10- to 25-km) to better reflect the running population at large. However, the runners diagnosed with EAH in this study were those participating in longer (42-, 84-, and 100-km) races, supporting previous data that reported longer distances and longer exercise duration as established risk factors for developing EAH [16, 29]. Despite a similar percentage of runners who were overhydrated in the shorter race distances (10- to 25-km), the lack of documented, symptomatic, EAH could possibly result from either an insufficient time for EAH symptoms to manifest or the runners’ preference to “endure it through” rather than report to the medical tent immediately following race.

Few studies on EAH have been performed in the Asian context [14], and this study adds to the existing literature by providing figures for the prevalence of symptomatic EAH in a tropical climate where temperature and humidity are generally higher [10, 14] compared to the Western context. According to the study by Hoffman et al. [10], races with higher ambient temperatures were associated with the highest incidences of EAH. This suggests that countries that experience higher ambient temperatures all year round could be at greater risk of EAH, underscoring the importance of knowledge regarding EAH in these climates. Hoffman et al. [10] also reported that EAH with dehydration was more predominant than EAH with overhydration in races with higher ambient temperatures. The prevalence of overhydration, based on percent changes in body mass, of 6 % in our study was much lower compared to the figure of 34.9 % quoted in the study by Hoffman et al. [10] This disparity is however more likely to be a result of a combination of factors including race distance, interval of fluid stations, racing speed, and other confounding factors in addition to the ambient temperatures alone.

Female gender has also been found to be a risk factor for EAH, with more dire symptomatology and outcomes with equivalent levels of hyponatremia [29, 30]. The underlying etiology remains unclear, but some studies have suggested that estrogen increases ADH secretion, lowers the thirst threshold, and blunts the drive to consume sodium [31, 32]. Other than estrogen, another factor contributing to female gender being a risk factor for overhydration may possibly be a differential behavior towards hydration. This is a factor that has yet to be explored more critically. However, Almond et al. [11] demonstrated that even though the incidence of EAH is higher in women, there were no statistical significance differences after adjusting for race time and BMI. These findings were similarly reflected in our study, where the incidence of overhydration (7 % in women versus 6 % in men) and EAH (0.3 % in women versus 0.5 % in men) was similar. We recognize, however, that the lower percentage of women (19 %) in our study sample could have led to the underreporting of results. We thereby propose to conduct longitudinal studies in the future with the inclusion of more women for more balanced gender analyses.

Various strategies have been proposed to reduce the occurrence of EAH, but the primary preventive strategy remains avoidance of overdrinking during a race. However, the threshold for overdrinking is often difficult to define in practice. Current recommendations suggest that fluid intake be based on the sensation of thirst because it reduces both the risk of dehydration and overhydration [3]. Reducing the availability of fluid stations at more than 3 km apart was also shown to be effective in reducing the incidence of EAH [3, 33]. More importantly, information on fluid balance should be disseminated to the runners to educate them on the current recommendations and increase the awareness of EAH. Outdated advice such as “drink beyond thirst” should be corrected. This strategy has been shown to be similarly effective in various studies [33, 25]. Other strategies proposed include placement of on-site weighing scales and sodium supplements. While usage of weighing scales would allow runners to screen for post-race body mass gain and seek early medical help, the absence of it does not exclude EAH as body mass loss has been reported in some cases of runners with EAH [10]. The usage of sodium supplements remains controversial. While sodium ingestion during a race may attenuate the fall in blood sodium concentrations, when body mass losses are fully replaced, it does not prevent the development of EAH if overdrinking continues [34, 35]. Current literature also recommends the avoidance of excessive sodium intake [3]. Therefore, the cornerstone in the prevention of EAH remains the prevention of overdrinking.

There are a few limitations worth noting in this study. First, the amount of fluid consumed during the race was not determined, as these data were too logistically challenging to collect. Second, initial hydration status was not assessed before the race and a small proportion of runners may have started either dehydrated or overhydration. Third, only symptomatic runners were admitted to the on-site medical tent and had their blood sodium levels measured; thus, our numbers do not represent the exact prevalence of EAH. Their body mass changes could not be evaluated, as they were incapable of standing on the weighing scale. And finally, the relationship between changes in body mass may not accurately reflect changes in total body water in field settings although runners should never gain body mass (indicative of overhydration) during exercise.

## Conclusions

Approximately 3–8 % runners had increased post-race BM, suggesting overhydration regardless of race distance. Symptomatic EAH was seen at race distances at or above 42-km. It is hoped that our study will encourage and provide direction to relevant authorities and stakeholders to develop a more rational and pragmatic fluid replacement strategy, aimed at optimizing, rather than maximizing fluid intake.
